# Advances in clear aligner therapy: comparative evaluation of the optical properties and bacterial adhesion of 3D direct-printed and thermoformed aligners

**DOI:** 10.1186/s40510-025-00600-3

**Published:** 2025-12-18

**Authors:** Daniel De-Shing Chen, Tzu-Yu Peng, Pin-Yu Huang, Masato Kaku, Johnson Hsin-Chung Cheng

**Affiliations:** 1 School of Dentistry, College of Oral Medicine, Taipei Medical University, Taipei, Taiwan; 2 Division of Orthodontics, Department of Oral Medicine, Taipei Medical University Hospital, Taipei, Taiwan; 3 Department of Anatomy and Functional Restorations, Graduate School of Biomedical and Health Sciences, Hiroshima University, Hiroshima, Japan

**Keywords:** Clear aligners, 3D printing, Thermoformed materials, Surface roughness, Color stability, Light transmittance, Bacterial adhesion

## Abstract

**Background:**

Clear aligner therapy is gaining traction owing to its esthetics and comfort. Although most aligners use thermoforming, 3D printing offers advantages such as higher accuracy and reduced waste. While literature on the properties of some 3D-printed aligner materials compared to thermoformed ones is available, a comprehensive study is currently lacking that compares all three materials (iLuxclear (LC), Graphy Clear Aligner (GY), and RightBio Clear Aligner (RD)) with thermoformed materials, particularly regarding optical and biofilm adhesion characteristics.

**Methodology:**

Three 3D direct-printed materials (LC, GY and RD) and two thermoformed materials (easyDu (ED) and Biolon (SC)) were tested. Surface morphology was analyzed by stereomicroscopy. Surface roughness (Ra) was measured at baseline (0 day) and after 45 days of immersion in artificial saliva. Light transmittance and color stability (ΔE_00_) were evaluated after 7 and 14 days of aging in saliva, black tea, and coffee. Bacterial adhesion was quantified using *Streptococcus mutans* (*S. mutans*) at baseline and after 3 and 7 days.

**Results:**

The 3D direct-printed aligners, particularly the LC group, exhibited increased surface morphology irregularities and significantly higher Ra values than the thermoformed materials; Ra increased after 45 days of immersion in artificial saliva across all groups. The thermoformed materials maintained stable color integrity, while the 3D-direct printed materials varied in performance. GY demonstrated a uniform surface structure, lower roughness, and the highest color stability, whereas LC and RD experienced significant discoloration. The RD group exhibited significantly higher *S. mutans* adhesion, whereas the thermoformed materials exhibited superior biofilm resistance. Notably, GY achieved comparable *S. mutans* adhesion to the thermoformed materials after a 7-day culture.

**Conclusions:**

Among the 3D direct printed aligners, GY achieved comparable surface and microbiological performance to conventional options. These findings underscore their potential for balancing esthetics, susceptibility to bacterial adhesion, and clinical performance in clear aligner therapy.

## Background

 In recent years, clear aligner therapy has emerged as a widely adopted alternative to conventional fixed braces owing to its esthetic appeal, improved comfort, flexible scheduling, ease of cleaning, and removability [1, 6, 12, 18, 22]. Conventionally, clear aligners are fabricated via thermoforming, where a heated plastic sheet is molded over a 3D dental model [10, 13]. However, with advances in additive manufacturing, 3D direct-printing has become a viable alternative [3, 4, 11, 14], eliminating the thermoforming step and offering advantages such as higher dimensional accuracy, customizable thickness, and reduced material waste [5, 11, 15].

Cole et al. [5] investigated fit and dimensional stability of aligners fabricated using both methods. Although 3D direct-printed retainers exhibited greater variations on smooth surfaces, their fit at incisal and cusp tip regions was comparably to vacuum-formed retainers. Kim et al. [11] reported superior accuracy for direct printed aligners than thermoformed aligners. Similar studies [4, 18] have shown variability in material properties of the aligners. Additionally, recent studies have highlighted the potential of 3D printed aligners using shape memory polymers, which exhibit temperature-activated shape recovery properties in the oral environment, and offer improved adaptability and sustained orthodontic forces [3].

As demand for clear aligner therapy grows, an increasing number of patients are selecting this treatment modality, placing more emphasis on esthetics and maintaining oral health. Nevertheless, these advantages necessitate a critical evaluation of the physical and mechanical performance of direct-printed aligners, particularly when compared to thermoformed ones in terms of durability, flexibility, and optical stability [8, 20]. Accordingly, this study makes a novel contribution to the literature by evaluating the surface roughness, light transmittance, color stability, and biofilm formation of both 3D direct-printed and thermoformed clear aligners. The null hypothesis states that the manufacturing method—3D direct printing or thermoforming—does not result in significant differences in surface characteristics, optical properties, or bacterial adhesion.

## Methods

### Experimental sample preparation

Plate-shaped samples (10 mm × 10 mm) with a thickness of 0.75 mm were prepared from five different clear aligner materials, comprising three 3D direct-printed and two thermoformed types, as summarized in Table 1. The samples were not surface-treated but were cleaned with 70% ethanol and air-dried prior to testing. Note that all following experimental procedures were performed by a single, well-trained operator to minimize inter-operator variability; statistical analysis and data collation were conducted independently by a second researcher to maintain data quality and credibility.

An a priori power analysis was performed using G*Power software (version 3.1.9.6; Heinrich Heine University, Düsseldorf, Germany) to determine the appropriate sample size. Surface roughness, light transmittance, and color stability were planned with a repeated-measures ANOVA; biofilm was measured on independent samples, with power based on between-subjects one-way ANOVA at each time point. Under Cohen’s f = 0.40 and α = 0.05, a sample size of *n* = 12 achieves power = 0.80.

**Table 1:** Materials used in the current study

Identification	Manufacturer	Processed method	Abbr.
Dental Clear Aligner	LuxCreo Inc., Chicago, IL, United States	3D direct-printed	LC
Tera Harz Clear TC85	Graphy Co., Ltd., South Korea	3D direct-printed	GY
Right Appliance	RightDent Medtech Co., Ltd., Taiwan	3D direct-printed	RD
easyDu	BenQ Materials Corp., Taiwan	Thermoformed	ED
Biolon	SCHEU-DENTAL GmbH, Germany	Thermoformed	SC

Abbr. is the abbreviation used for each material in this study

### Surface morphology

All samples were examined using a stereo microscope (VMD-0745(500), OCCA; High-Accu. Trading Co., Ltd., Taiwan) at 30× magnification. Images were acquired using the built-in digital camera and calibrated with a stage micrometer. Observation conditions, including illumination intensity and aperture settings, were kept constant, with only linear adjustments made to brightness and contrast.

### Surface roughness

The samples were subjected to two aging conditions: no immersion (I_0_) and immersion in artificial saliva at 37 °C for 45 days (I_45_), simulating approximately four years of intraoral clinical use. Surface roughness (Ra, µm) was subsequently measured using a contact profilometer (Surftest SJ-210, Mitutoyo Corp., Japan). For each sample, five randomly selected linear paths of 6.4 mm were scanned at a speed of 0.5 mm/s. To ensure consistency, mean Ra values were calculated from five measurements per sample.

### Artificial color aging procedure

To simulate staining and aging in the oral environment, samples were immersed in three different solutions: artificial saliva (AS), black tea (BT), and coffee (CF). All solutions were kept at 37 °C throughout the immersion period and refreshed every three days to ensure stability. The samples were divided into three subgroups according to the duration of immersion: 0 (T_0_), 7 (T_7_), and 14 days (T_14_). At each designated time point, the samples were removed from the solution, rinsed with distilled water, and dried in air.

### Light transmittance

After artificial color aging at each time point (T_0_, T_7_, and T_14_), the light transmittance of each sample was measured using a haze meter (YH1200, Shenzhen ThreeNH Technology Co., Ltd., China) under standard illuminant C conditions according to the guidelines of ASTM D1044 guidelines. Each sample was positioned vertically in the sample holder and the percentage of transmitted light was recorded.

### Color stability

Color changes were assessed using a digital colorimeter (Optishade Styleitaliano; Smile Line SA, Switzerland) based on the CIE Lab* color space. The measurements were taken at the baseline (T_0_) and after 7 and 14 days of immersion (T_7_ and T_14_, respectively) following the artificial color aging procedure. The color differences (ΔE₀₀) between two time point (T_7_, and T_14_) and baseline (T_0_) were calculated using the following CIEDE2000 formula according to the official CIE technical report [9]:

$$ \begin{gathered} \Delta E_{{00}} \hfill \\ = \sqrt {~\left( {\frac{{\Delta L^{\prime}}}{{k_{L} ~S_{L} }}} \right)^{2} + \left( {\frac{{\Delta C^{\prime}}}{{k_{C} ~S_{C} }}} \right)^{2} + \left( {\frac{{\Delta H^{\prime}}}{{k_{H} ~S_{H} }}} \right)^{2} + R_{T} \left( {\frac{{\Delta C^{\prime}}}{{k_{C} ~S_{C} }}} \right)\left( {\frac{{\Delta H^{\prime}}}{{k_{H} ~S_{H} }}} \right)~} \hfill \\ \end{gathered} $$

where ΔLʹ, ΔCʹ, and ΔHʹ represent differences in brightness, chroma, and hue, respectively; k_L_, k_C_, and k_H_ are the weighting factors; S_L_, S_C_, and S_H_ are the corresponding scaling functions; and R_T_ is the rotation term that accounts for the interaction between chroma and hue differences.

In addition, the ΔE values were converted to National Bureau of Standards (NBS) units using the formula: NBS = ΔE×0.92. The NBS system classifies color changes into clinically meaningful categories: trace (0.0–0.5), slight (0.5–1.5), noticeable (1.5–3.0), appreciable (3.0–6.0), much (6.0–12.0), and very much (≥ 12.0). These categories correspond to increasing levels of perceptibility and provide a more intuitive interpretation of color changes compared to solely ΔE values [2, 7]. 

### Bacterial adhesion (biofilm)

Samples were disinfected by immersion in 75% ethanol for 5 min and then air-dried overnight. Each sample was inoculated with 1 mL of *Streptococcus mutans* (*S. mutans*, BCRC 10793; Bioresource Collection and Research Center, Hsinchu, Taiwan) suspension (1 × 10 CFU/mL) prepared in brain-heart infusion broth and incubated anaerobically at 37 °C for either 3 or 7 days. After incubation, non-adherent bacteria were removed by carefully rinsing the samples with phosphate-buffered saline (PBS). All the samples were fixed in 10% neutral-buffered formalin for 3 h at room temperature. After fixation, the samples were rinsed with PBS and incubated for 30 min. Biofilm staining was performed with a 0.1% crystal violet solution for 30 min, followed by another rinse with PBS and incubation for 30 min. To quantify the biofilm biomass, the retained crystal violet stain was dissolved with acetic acid for 10 min, and the absorbance was measured at 490 nm (OD₄₉₀).

### Statistical analysis

Statistical analyses were performed using GraphPad Prism (ver.10; GraphPad Software, San Diego, CA, United States; RRID: SCR_002798). The Shapiro–Wilk test was used to assess the normality of the data distribution, and Levene’s test was applied to evaluate the homogeneity of variances. Surface roughness was analyzed by one-way repeated-measures analysis of variance (ANOVA) by immersion time. Light transmittance and color stability used two-way repeated-measures ANOVA with solution and immersion time as within-subject factors. Biofilm outcomes used one-way ANOVA across materials per time point. Tukey’s honest significant difference test was applied for post-hoc comparisons, with two-sided *P* < 0.05 significant.

## Results

**Fig. 1:**
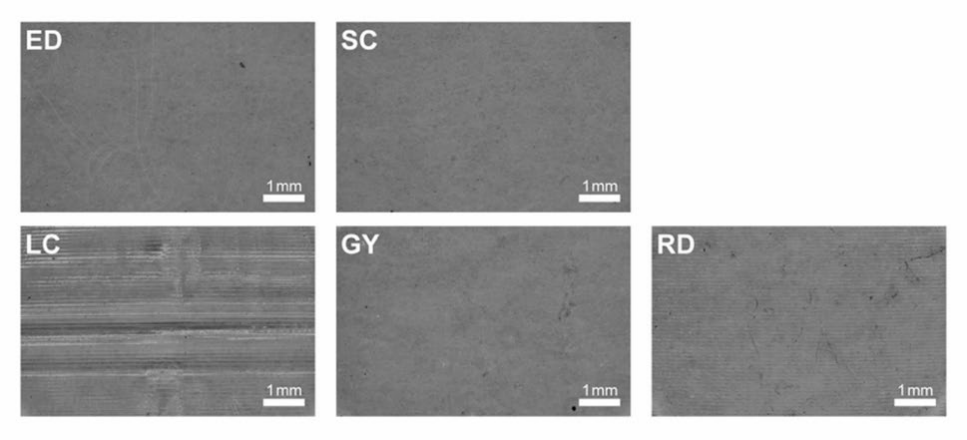
Stereomicroscopic observations of surface morphology

The stereomicroscopic analysis of surface morphology (Fig. 1) revealed that the thermoformed materials (ED and SC) exhibited relatively smooth surfaces. Among the 3D direct-printed materials, GY displayed surface characteristics comparable to those of the thermoformed materials. In contrast, LC and RD showed pronounced horizontal striations, with LC showing the most prominent features.

**Fig. 2:**
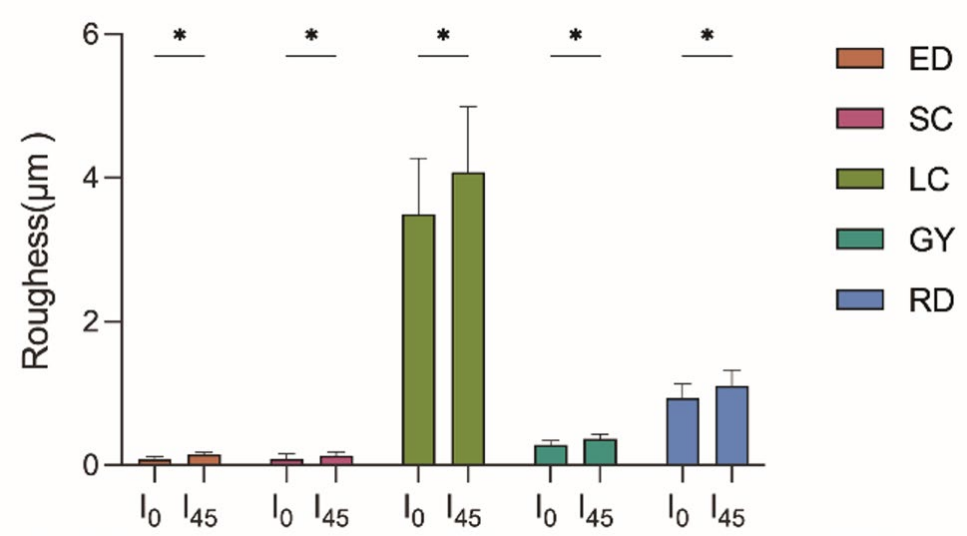
Results of the surface roughness (Ra). An asterisk (*) indicates a statistically significant difference among groups (*P* < 0.05)

The results of surface roughness (Ra) are shown in Fig. 2. The LC group showed the highest Ra value (*P* < 0.05), followed by the RD and GY groups. In particular, the thermoformed materials (ED and SC) had significantly lower Ra values (*P* < 0.05), although there were no significant differences between them. In addition, all materials showed a significant increase in Ra after 45 days of immersion in artificial saliva (*P* < 0.05).

**Fig. 3:**
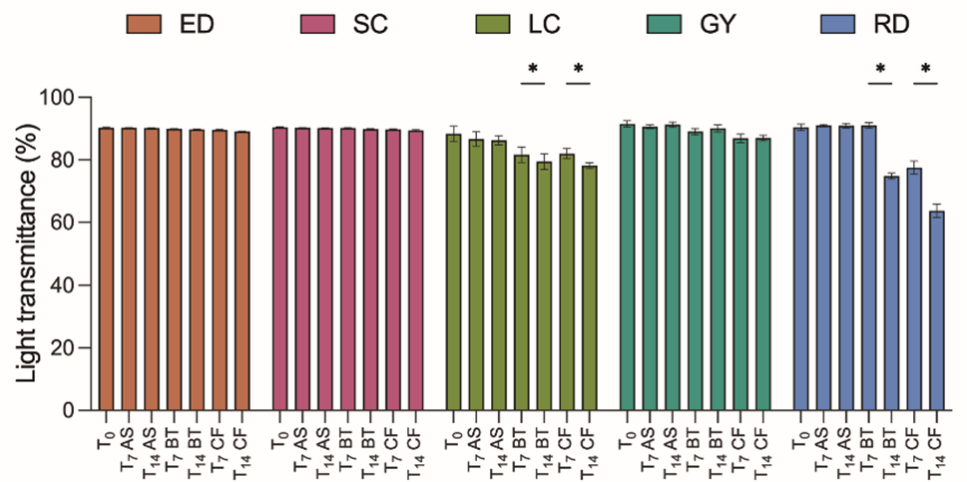
Light transmittance results. Samples were immersed in three different solutions—artificial saliva (AS), black tea (BT), and coffee (CF)—for 7 days (T_7_) and 14 days (T_14_). The asterisk (*) denotes a statistically significant difference (*P* < 0.05) between groups

Figure 3 shows the light transmittance values of the individual materials. All materials exhibited a decreasing trend in transmittance after immersion. The thermoformed materials (ED and SC) showed relatively stable results, whereas the 3D direct-printed materials (LC, GY, and RD) exhibited significant variations (*P* < 0.05). Among them, GY showed the most stable performance with no significant changes regardless of the solution type or immersion period. By contrast, both LC and RD showed a significant decrease in transmittance after immersion in black tea and coffee, with RD showing the largest decrease (*P* < 0.05).

Table 2 lists the ΔE and corresponding NBS values. The thermoformed materials (ED and SC) showed relatively stable results, with most ΔE values remaining below 1.5. By contrast, the 3D direct-printed materials showed significantly greater variation (*P* < 0.05). Among them, GY demonstrated the highest color stability, with all ΔE values remaining below 6.0. LC and RD exhibited significant discoloration after immersion in black tea and coffee, with RD showing the greatest change (ΔE > 17.0 at T_14_ in coffee; *P* < 0.05). LC also showed marked discoloration in black tea (ΔE > 6.5) and coffee (ΔE > 13.6) (*P* < 0.05). Significant differences between T_7_ and T_14_ were observed in most groups, particularly in the coffee group (*P* < 0.05). According to NBS, ED and SC remained within the “slight” level (0.5–1.5). RD reached the “very much” level (≥ 12.0) after coffee immersion, while LC fell into the “appreciable” to “very much” level. Nevertheless, GY remained consistently at a “noticeable” to “appreciable” level under all conditions (NBS < 6.0).

**Table 2:** Color differences (ΔE) and corresponding National bureau of standards (NBS) units

Group		*ED*		*SC*		*LC*		*GY*		*RD*	
		ΔE	NBS	ΔE	NBS	ΔE	NBS	ΔE	NBS	ΔE	NBS
AS	T_7_	0.78 ± 0.30	0.72 ± 0.27	1.20 ± 0.28	1.11 ± 0.26	1.45 ± 1.47	1.34 ± 1.35	1.28 ± 0.42	1.17 ± 0.39	0.60 ± 0.35	0.55 ± 0.33
	T_14_	1.16 ± 0.65	1.06 ± 0.59	0.99 ± 0.42	0.91 ± 0.39	1.86 ± 0.18	1.71 ± 0.16	0.96 ± 0.16	0.88 ± 0.15	0.59 ± 0.31	0.55 ± 0.29
BT	T_7_	1.00 ± 0.26	0.92 ± 0.24	0.99 ± 0.26	0.91 ± 0.24	5.70 ± 1.34^*^	5.24 ± 1.23^*^	1.26 ± 0.23	1.16 ± 0.21	10.33 ± 1.04^*^	9.50 ± 0.95^*^
	T_14_	0.98 ± 0.54	0.90 ± 0.49	1.03 ± 0.43	0.95 ± 0.39	6.89 ± 1.15^*^	6.34 ± 1.06^*^	1.50 ± 0.32	1.38 ± 0.30	13.91 ± 0.64^*^	12.8 ± 0.59^*^
CF	T_7_	0.73 ± 0.37^*^	0.67 ± 0.34^*^	1.20 ± 0.36	1.11 ± 0.33	9.74 ± 0.66^*^	8.96 ± 0.61^*^	3.70 ± 0.73^*^	3.40 ± 0.67^*^	14.47 ± 0.61^*^	13.31 ± 0.56^*^
	T_14_	1.45 ± 0.44^*^	1.34 ± 0.41^*^	1.45 ± 0.45	1.33 ± 0.41	13.57 ± 0.63^*^	12.48 ± 0.58^*^	5.66 ± 1.07^*^	5.21 ± 0.99^*^	17.00 ± 0.42^*^	15.64 ± 0.38^*^

Samples were immersed in three different solutions—artificial saliva (AS), black tea (BT), and coffee (CF)—for 7 days (T_7_) and 14 days (T_14_), and the corresponding color differences (ΔE) from the baseline (T_0_) were calculated. The asterisk (^*^) denotes a statistically significant difference (*P* < 0.05) between T_7_ and T_14_ for the same material and solution. The classification of NBS levels: trace (0.0–0.5), slight (0.5–1.5), noticeable (1.5–3.0), appreciable (3.0–6.0), much (6.0–12.0), and very much (≥ 12.0)

**Fig. 4:**
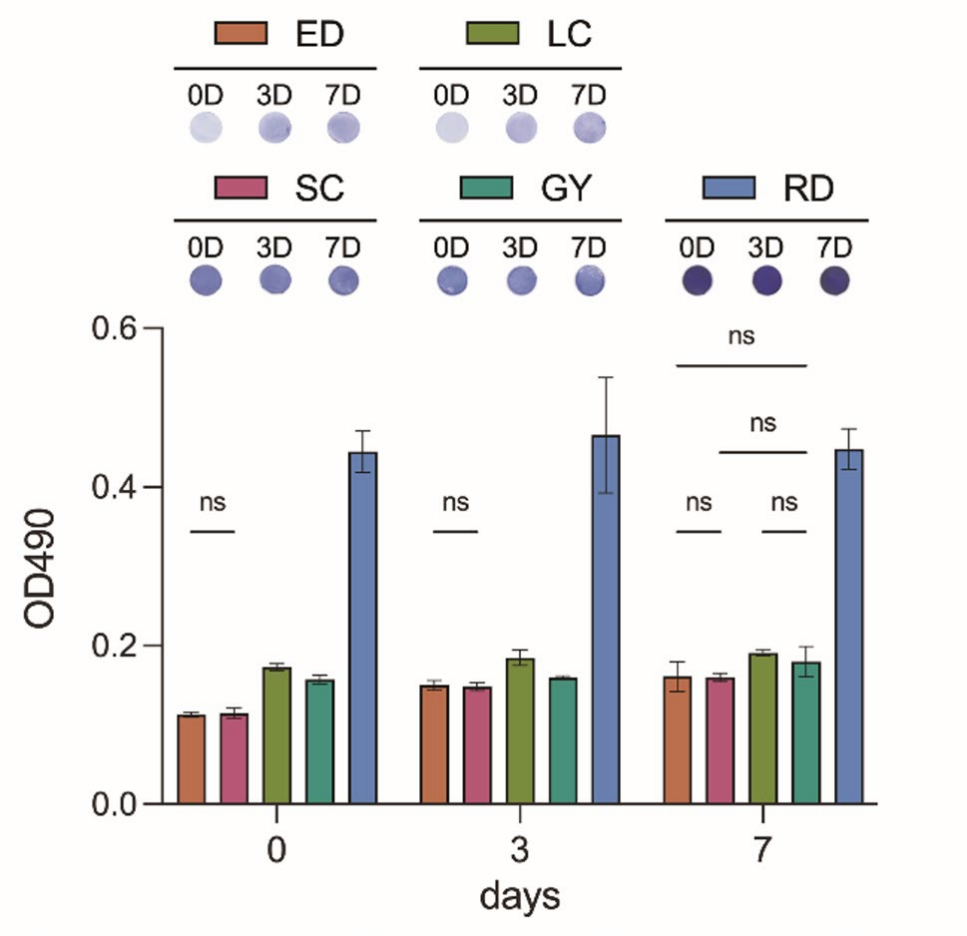
Biofilm formation results. “ns” denote no statistically significant differences within groups (*P* > 0.05)

Figure 4 illustrates the OD490 values representing biofilm formation after 0, 3, and 7 days. At all time points, the RD group consistently exhibited the highest OD490 values. No statistically significant differences were observed between the two thermoformed materials (ED and SC) at any time point (ns; *P* > 0.05). At baseline (day 0) and on day 3, all the 3D direct-printed materials (LC, GY, and RD) showed significantly higher OD490 values than the thermoformed materials. On day 7, the OD490 value of GY no longer differed significantly from that of ED and SC (ns, *P* > 0.05), whereas the values of LC and RD remained significantly different (*P* < 0.05).

## Discussion

The advent of 3D direct-printed aligners represents a significant advancement in clear aligner therapy [3, 4, 14, 20]. By reducing fabrication steps and costs while enhancing design flexibility, this technology presents a compelling alternative to conventional thermoformed aligners [8, 15, 20]. In this study, the null hypothesis was partially rejected: although thermoformed aligners generally outperformed their 3D counterparts, certain 3D direct-printed aligners demonstrated comparable performance. These findings suggest that, although thermoformed aligners are advantageous in several aspects, 3D direct-printed aligners may offer clinically acceptable alternatives in terms of specific properties, expanding the range of viable material options for clear aligners.

Surface roughness significantly influences both esthetic outcomes and bacterial adhesion to aligner surfaces [16, 21]. In this study, the directly printed aligners exhibited significantly higher baseline surface roughness than the thermoformed materials, with LC showing the highest Ra values (Fig. 2). All materials exhibited increased surface roughness following artificial saliva aging, simulating long-term intraoral use. Increased roughness has been associated with plaque accumulation and discoloration, which negatively impact oral hygiene and esthetics. These findings are consistent with previous studies, suggesting that the additive layer-by-layer nature of 3D printing contributes to more uneven surface morphology than the uniform surface produced via thermoforming [4, 11]. It should be noted that, for surface roughness measurements, the samples were immersed in artificial saliva for 45 days; this setting was used to characterize early post-processing surface stabilization and potential changes and served only as a conservative upper bound to ensure signal stabilization and detect late-emerging effects, and was not intended to align with the clinical replacement cycle.

The thermoformed aligner materials outperformed their 3D direct-printed counterparts in terms of maintaining translucency and minimizing bacterial adhesion (Fig. 4). Among the 3D direct-printed groups, GY exhibited significantly smoother (Fig. 2) and more uniform surface morphology (Fig. 1) than the other two materials (*P* < 0.05).

Optical properties, such as transmittance and color stability, are crucial to meet the growing demand for discreet and esthetically pleasing orthodontic appliances. GY, a 3D direct-printed aligner, maintained stable transmittance (Fig. 3) and color appearance (Table 2) across the immersion conditions, whereas LC and RD showed significant discoloration (*P* < 0.05), particularly with coffee and black tea. Thermoformed materials were more resistant to discoloration and exhibited less color change, generally remaining within the “slight” level according to the NBS classification (Table 2). These results support previous observations that the composition of the aligner material, porosity, and surface integrity influence resistance to color aging [4]. Notably, although 3D direct-printed materials typically exhibit high variability, GY emerged as a promising exception, likely owing to its unique polymer formulation and printing precision.

Biofilm accumulation, governed by surface texture and hydrophobicity, is critical for maintaining oral health during clear aligner therapy. The RD group consistently showed the highest levels of bacterial adhesion, whereas the thermoformed aligners demonstrated superior resistance. Notably, GY performed comparably to the thermoformed materials until day 7, indicating that not all 3D direct-printed materials are equally susceptible to bacterial colonization (Fig. 4). These differences are likely attributed to variations in polymer chemistry and surface properties post-curing. Given the increasing importance of hygiene in orthodontic care, these findings reinforce the need for further innovation in 3D printing materials to reduce microbial risks [17, 19, 21].

Although 3D direct-printed aligners still encounter limitations in esthetics and surface roughness compared with thermoformed materials, notable progress has been made. Several limitations remain in the current study. The immersion time used for the surface roughness test does not match the clinical wear period of clear aligners; future studies will adopt clinically aligned follow-up points to better reflect real-world conditions and enhance external validity. In addition, current work also did not quantify material-specific water sorption and solubility under oral-like conditions or assess ion release and chemical/functional-group changes. Subsequent work should couple surface-topography changes with chemical signatures and leachable profiles to strengthen inferences about clinical risk and durability. Within the limitations of this study, the GY material exhibited a surface roughness level comparable to that of the thermoformed aligners and showed no statistically significant increase in plaque accumulation. These findings indicate that, with appropriate material selection, 3D direct-printed aligners can achieve comparable performance to that of conventional aligners in key clinical performance metrics. Clinicians and researchers must continue to monitor material-specific outcomes when using this innovative approach. This study provides valuable guidance for the selection of 3D direct-printed and thermoformed materials in terms of esthetic demands and susceptibility to bacterial adhesion for clear aligner therapy.

## Conclusions

The conclusions of this study can be summarized as follows:

1. The 3D direct-printed materials exhibited a higher baseline surface roughness than the thermoformed control materials. However, all materials showed a significant increase after 45 days of immersion in artificial saliva, indicating time-dependent changes across all modalities rather than a modality-specific limitation.
2. Among the direct-printed resins, GY demonstrated the most stable optical performance, with no significant change in transmittance in any solution and with all ΔE values < 6 (NBS “noticeable–appreciable”), comparable to thermoformed materials. By contrast, LC and RD resulted in pronounced discoloration of black tea and coffee, respectively.
3. GY showed higher initial biofilm formation than the thermoformed materials; however, no significant difference was observed by day 7, whereas LC and RD maintained elevated levels, with RD consistently exhibiting the highest levels. These results indicate that appropriately selected 3D direct-printed materials, particularly GY, can achieve microbiological performance comparable to thermoformed.

## Data Availability

The datasets used and/or analysed during the current study are available from the corresponding author on reasonable request.
